# The Prognosis and Immune Checkpoint Blockade Efficacy Prediction of Tumor-Infiltrating Immune Cells in Lung Cancer

**DOI:** 10.3389/fcell.2021.707143

**Published:** 2021-08-03

**Authors:** Xiangzheng Liu, Xueqian Shang, Jian Li, Shijie Zhang

**Affiliations:** Department of Thoracic Surgery, Peking University First Hospital, Peking University, Beijing, China

**Keywords:** lung cancer, B cell, myeloid dendritic cell, prognosis, immune checkpoint blockade

## Abstract

**Backgrounds:**

The high morbidity and mortality of lung cancer are serious public health problems. The prognosis of lung cancer and whether to apply immune checkpoint blockade (ICB) are currently urgent problems to be solved.

**Methods:**

Using R software, we performed Kaplan–Meier (K-M) analysis, Cox regression analysis, functional enrichment analysis, Spearman correlation analysis, and the single-sample gene set enrichment analysis.

**Results:**

On the Tumor IMmune Estimation Resource (TIMER2.0) website, we calculated the abundance of tumor-infiltrating immune cells (TIICs) of lung adenocarcinoma (LUAD) and lung squamous cell carcinoma (LUSC) patients. B cell and myeloid dendritic cell (DC1) were independent prognostic factors for LUAD and LUSC patients, respectively. Enrichment analysis confirmed that genes highly related to B cell or DC1 were closely related to the immune activation of lung cancer patients. In terms of adaptive immune resistance markers, CD8A, CD8B, immunomodulators (immunostimulants, major histocompatibility complex, receptors, and chemokines), immune-related pathways, tumor microenvironment score, and TIICs, high B cell/DC1 infiltration tissue was inflamed and immune-activated and might benefit more from the ICB. Genes most related to B cell [CD19, toll-like receptor 10 (TLR10), and Fc receptor-like A (FCRLA)] and DC1 (ITGB2, LAPTM5, and SLC7A7) partially clarified the roles of B cell/DC1 in predicting ICB efficacy. Among the 186 Kyoto Encyclopedia of Genes and Genomes (KEGG) pathways, there were three and four KEGG pathways, which partially explained the molecular mechanisms by which B cell and DC1 simultaneously predicted the prognosis and efficacy of immunotherapy, respectively. Among five immune subtypes, the abundance of B cell/DC1 and expression of six hub genes were higher in immune C2, C3, and C6.

**Conclusion:**

B cell and DC1 could predict the prognosis and ICB efficacy of LUAD and LUSC patients, respectively. The six hub genes and seven KEGG pathways might be novel immunotherapy targets. Immune C2, C3, and C6 subtypes of lung cancer patients might benefit more from ICB therapy.

## Introduction

According to Global Cancer Statistics 2020, lung cancer is the most common cause of cancer-related deaths worldwide, whose incidence rate ranks second (second only to breast cancer). Specifically, in 2020, there were 2.2 million new cases and 1.8 million new deaths (Sung et al., 2021). Non-small cell lung cancer (NSCLC) is the main histologic subtypes (accounting for 85%) of lung cancer, of which approximately 50% are lung adenocarcinoma (LUAD) and approximately 30% are lung squamous cell carcinoma (LUSC) (Perez-Moreno et al., 2012).

The unoptimistic mortality rate makes lung cancer patients full of doubts about how long they can live, which is a problem that clinicians cannot avoid and facilitates the construction of various prognostic signatures (He and Zuo, 2019; Zuo et al., 2019). In terms of treatment, apart from traditional chemotherapy, targeted therapy, and antiangiogenesis therapy, novel immunotherapy based on programmed cell death ligand 1 (PD-L1, also called CD274)/ligand of programmed cell death 1 (PD-1), has attracted increasing attention. However, adhering to the principles of personalized medicine and precision medicine, which lung cancer patients might benefit more from immunotherapy, is a vital issue that urgently needs to be solved. Therefore, it is crucial to identify new targets or signatures that could predict both prognosis and immunotherapy efficacy of lung cancer patients.

In our study, based on the Tumor IMmune Estimation Resource (TIMER2.0), we quantified the abundance of tumor-infiltrating immune cells (TIICs) in the tumor microenvironment (TME) of The Cancer Genome Atlas (TCGA)-LUAD and TCGA-LUSC. Kaplan–Meier (K-M) and Cox regression analysis proved that B cell and myeloid dendritic cell (DC1) were independent prognostic factors for LUAD and LUSC patients, respectively. The functional enrichment analysis of genes highly related to B cell or DC1 (Spearman correlation coefficient >0.6) in lung cancer focused on B cell activation and T cell activation, respectively, indicating that the above two prognostic factors were significantly related to the activation of immune system of lung cancer. In terms of the expression of adaptive immune resistance markers, CD8A, CD8B, immunomodulators [immunostimulants, major histocompatibility complex (MHC), receptors, and chemokines], immune-related pathways, and TIICs, compared with tumor tissues in the low B cell/DC1 infiltration group, most immune biomarkers were significantly upregulated in the high B cell group (LUAD) or high DC1 group (LUSC). The above results indicated that high B cell/DC1 infiltration could shape an inflamed and immune-activated TME. We concluded that this part of lung cancer patients might benefit more from immune checkpoint blockade (ICB) therapy.

For the convenience of clinical application, we then obtained genes most related to B cell [CD19, toll-like receptor 10 (TLR10), and FCRLA] or DC1 (ITGB2, LAPTM5, and SLC7A7). In pan-cancer, we confirmed that they were significantly related to CD8A, CD8B, and PD-L1. The protein–protein interaction (PPI) functional enrichment results were significantly related to the activation and proliferation of immune cells, which further confirmed that six hub genes were significantly related to the activation of immune system. Given the overall TME scores of LUAD and LUSC patients, B cell, DC1, and six hub genes, like CD8A, CD8B, and PD-L1, were significantly positively correlated with stromal score and immune score and significantly negatively correlated with tumor purity. Besides, compared with the ICB non-response group (NR), the response group (R) had higher expression of six hub genes and abundance of B cell and DC1. Among five immune subtypes of lung cancer, immune C2, C3, and C6 of LUAD and LUSC might be easier to benefit from ICB treatment.

To further explore the molecular mechanisms of B cell and DC1 in predicting the prognosis and the efficacy of immunotherapy at the same time, among 186 Kyoto Encyclopedia of Genes and Genomes (KEGG) pathways, we mined three KEGG pathways in LUAD tissues, which clarified the mechanisms by which B cell predicted the prognosis and ICB response of LUAD patients. Similarly, for LUSC patients, we investigated four KEGG pathways, elucidating the molecular pathways of DC1 predicting the overall survival (OS) and ICB reactivity.

In conclusion, through these three KEGG pathways, B cell abundance predicted the OS and ICB response of LUAD patients. For LUCS patients, four KEGG pathways were the molecular mechanisms of DC1 that both predicted the prognosis and the ICB efficacy. The above seven KEGG pathways were expected to become new immunotherapy targets. Among the five immune subtypes of lung cancer patients, immune C2, C3, and C6 subtypes might benefit more from ICB therapy.

## Materials and Methods

### Data Source

The transcriptome expression profiles and clinical information of LUAD and LUSC were downloaded from TCGA^1^ and Gene Expression Omnibus (GEO) database^2^. Fragment per kilobase of transcript per million mapped reads (FPKM) was converted to TPM (transcript per kilobase of exon model per million mapped reads) and used in this study. For TCGA-LUAD, 512 LUAD tissues and 58 adjacent normal tissues were included in our study. In terms of TCGA-LUSC, there were 497 LUSC samples and 49 LUSC adjacent normal samples. For GSE31210, there were 226 LUAD tissues and 20 normal tissues. For GSE157009, there were 249 LUSC tissues without normal samples. Specific clinical information of LUAD and LUSC patients is shown in Table 1.

**TABLE 1:** The clinical characteristics of lung cancer patients in our study.

Variables	TCGA LUAD	TCGA LUSC	GSE31210 LUAD	GSE157009 LUSC
Total	512	497	226	249
Female (%)	276 (53.9)	129 (26.0)	121 (53.5)	88 (35.3)
Male (%)	236 (46.1)	368 (74.0)	105 (46.5)	161 (64.7)
Age (median [IQR])	66.00 [59.00, 73.00]	68.00 [62.00, 73.00]	61.00 [55.00, 65.00]	70.00 [64.00, 76.00]
**T (%)**				
NA	3 (0.6)	0 (0.0)		
T1	168 (32.8)	112 (22.5)		
T2	277 (54.1)	292 (58.8)		
T3	45 (8.8)	70 (14.1)		
T4	19 (3.7)	23 (4.6)		
**N (%)**				
NA	12 (2.3)	5 (1.0)		
N0	328 (64.1)	317 (63.8)		
N1	96 (18.8)	130 (26.2)		
N2	74 (14.5)	40 (8.0)		
N3	2 (0.4)	5 (1.0)		
**M (%)**				
NA	142 (27.7)	83 (16.7)		
M0	345 (67.4)	407 (81.9)		
M1	25 (4.9)	7 (1.4)		
**Stage (%)**				
NA	7 (1.4)	4 (0.8)		
Stage i	274 (53.5)	242 (48.7)	168 (74.3)	123 (49.4)
Stage ii	121 (23.6)	160 (32.2)	58 (25.7)	108 (43.4)
Stage iii	84 (16.4)	84 (16.9)		18 (7.2)
Stage iv	26 (5.1)	7 (1.4)		

### Quantification of TIICs

The TIMER2.0^3^ is a friendly platform for systematical evaluations of the clinical impact of different immune cells in diverse cancer types (Li et al., 2020). The abundance of TIICs, including B cell, CD4 T cell, CD8 T cell, macrophage, neutrophil, and DC1, was estimated on the 2.0 version of this website. Gene_Corr module is used to explore the correlation between interested genes with a list of genes in pan-cancer.

### The Prognostic Significance of Six Types of TIICs

Based on the median abundance of TIICs, we performed K-M analysis and log-rank test on the high- and low-infiltration groups. With the help of “survival” and “survminer” packages, we plotted survival curves. The Cox regression analysis was also included in our study and displayed in forest maps (In and Lee, 2019). Green represents univariate Cox analysis, whereas red represents multivariate Cox analysis.

### The Functional Enrichment Analysis

Among the transcriptome 19,464 protein-coding genes, taking the absolute Spearman correlation coefficient greater than 0.6 as the threshold (strong correlation) (Lin et al., 2016; Chen et al., 2017), we obtained genes that were highly related to B cell and DC1 in the TCGA-LUAD and TCGA-LUSC cohorts, respectively. Based on these highly related genes and “clusterProfiler” package, we performed Gene Ontology (GO) and KEGG analysis.

### The Single-Sample Gene Set Enrichment Analysis

The single-sample gene set enrichment analysis (ssGSEA) is an extension of GSEA and calculates a separate enrichment score for each sample. Each ssGSEA enrichment score represents the degree to which the genes in a particular gene set are coordinately upregulated or downregulated within a sample (Subramanian et al., 2005). In our study, based on the “GSVA” package (Hänzelmann et al., 2013) and gene sets [30 immune-related pathways (Shang et al., 2020) and 186 KEGG pathways], the ssGSEA score was used to explore the differences in the activation status of immune system between the high and low B cell/DC1 groups and mine the molecular mechanisms of B cell and DC1 in predicting prognosis and ICB efficacy. The above gene sets were downloaded from the MSigDB database^4^ (Liberzon et al., 2011).

### The GeneMANIA Database

The GeneMANIA^5^ is a database similar to STRING, based on which we can find genes with similar functions of interested genes and predict gene functions simultaneously (Warde-Farley et al., 2010).

### The Stromal and Immune Scores of the TME and Tumor Purity

Infiltrating stromal cells and immune cells constitute the main part of normal cells in tumor tissues, which not only disrupt tumor signals in molecular research, but also play crucial roles in cancer biology. The abundance of non-tumor cells (stromal cells and immune cells) and tumor purity of lung cancer tissues could be evaluated by incorporating two gene signatures (the stromal and immune signatures) using the ESTIMATE (using expression data to estimate stromal cells and immune cells in malignant tumor tissue) algorithm (Yoshihara et al., 2013). The stromal signature was used to capture stromal cells in the TME, whereas the immune signature was designed to represent the immune cell abundance in tumor tissues. The results of ESTIMATE algorithm were presented as immune score, stromal score, and ESTIMATE score. The higher the score, the greater the ratio of the corresponding component in the TME. Based on the ESTIMATE score, we further inferred tumor purity in tumor tissues.

### The Efficacy Prediction of ICB

On the Immune Cell Abundance Identifier (ImmuCellAI) website^6^, we predicted the ICB efficacy of lung cancer patients and divided them into the ICB response group and non-response group (Miao et al., 2020).

### Correlation Diagram and Heatmap

In our research, we plotted correlation diagrams using “corrplot” and “PerformanceAnalytics” packages. With the “pheatmap” package, we draw heatmaps. To generate the heatmaps, data were log2-transformed. Each column represents a sample, and each row represents one of the immunomodulators. The levels of immunomodulators are displayed in different colors, which transition from blue to red with increasing expression.

### Ethics Statement

All data in our study were obtained from the online public database, TCGA and GEO, and did not involve any *in vitro* or *in vivo* experiments.

### Statistical Analysis

All statistical analyses were performed in R software (version 4.0.3). As gene expression and immune cell abundance did not conform to the normal distribution, differences between two groups were tested by the Wilcoxon test, and differences between multiple groups were tested by the Kruskal–Wallis test. Based on the “ggstatsplot” package, the *P*-values were corrected for multiple testing using the Dunn test. *P* < 0.05 was considered statistically significant: ^∗^*p* < 0.05, ^∗∗^*p* < 0.01, ^∗∗∗^*p* < 0.001.

## Results

### The Survival Correlation of TIICs in Lung Cancer

**FIGURE 1:**
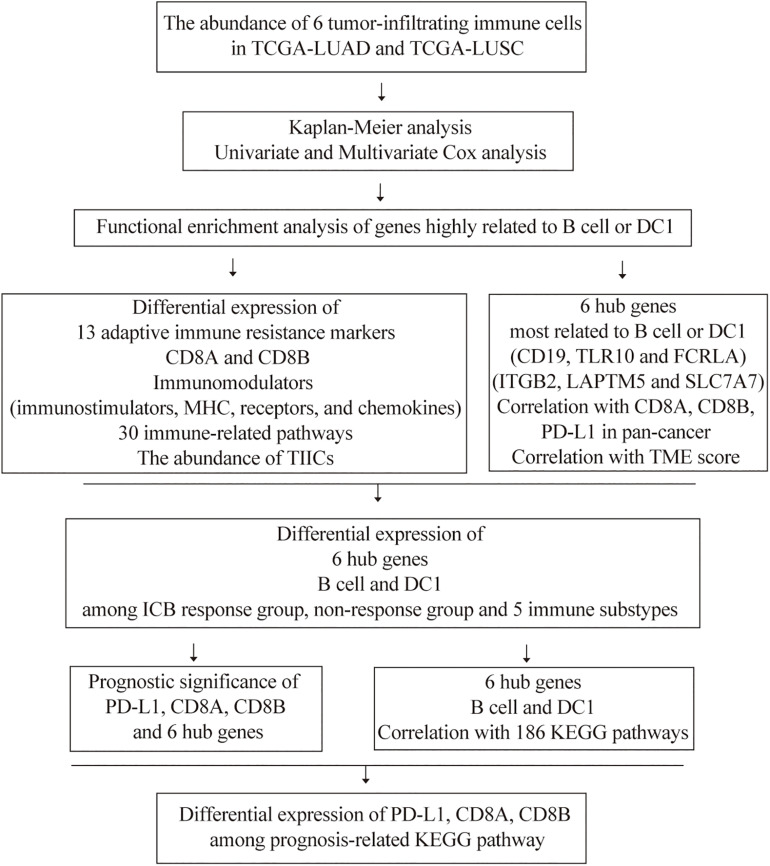
The flowchart of this study.

**FIGURE 2:**
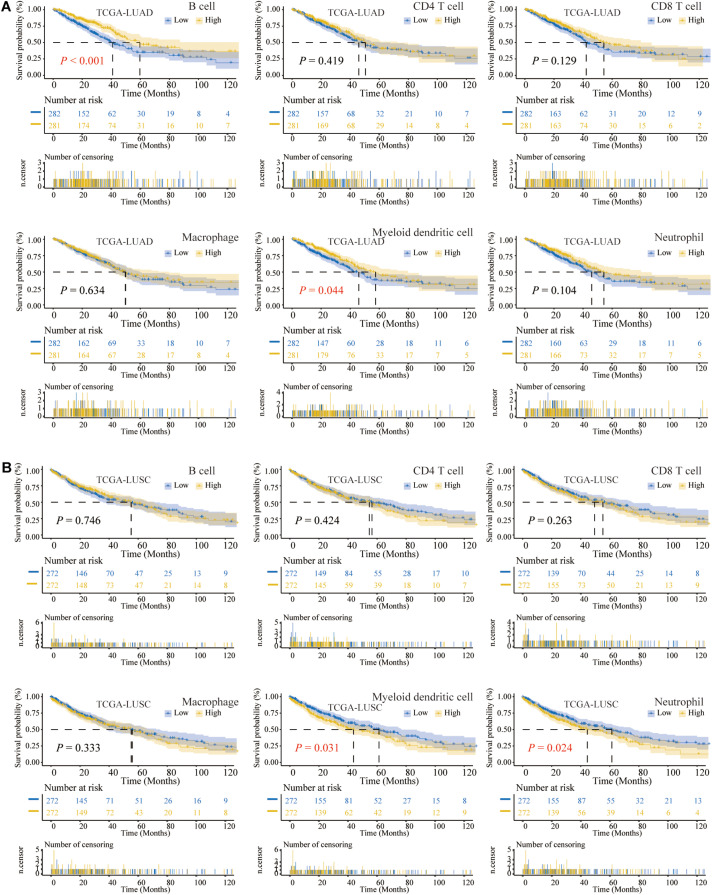
The Kaplan–Meier (K-M) analysis and log-rank test of six types of tumor-infiltrating immune cells (TIICs) in lung cancer. **(A)** For TCGA-LUAD, the higher the abundance of B cell or DC1, the longer the overall survival (OS). **(B)** In TCGA-LUSC, the lower the infiltration of DC1 or neutrophil, the longer the OS.

The flowchart of our study is shown in Figure 1. Previous studies had confirmed that the abundance of TIICs was closely related to the prognosis of cancer patients (Pagès et al., 2009; Waniczek et al., 2017). Based on the TIMER2.0, we quantified the abundance of TIICs in the TME of the TCGA-LUAD and TCGA-LUSC cohorts, including three types related to adaptive immunity: B cell, CD4 T cell, and CD8 T cell, as well as three types related to innate immunity: macrophage, DC1, and neutrophil. Among the above six TIICs, we performed K-M analysis and log-rank test. The OS of LUAD patients with high B cell or DC1 infiltration was longer than that of the low-infiltration group (*P* < 0.05) (Figure 2A), whereas compared with the high-infiltration group, LUSC patients with low DC1 or neutrophil infiltration had longer OS (*P* < 0.05) (Figure 2B). Therefore, B cell and DC1 were significantly related to the survival of LUAD patients, whereas DC1 and neutrophil were significantly related to the survival of LUSC patients.

### The Prognostic Significance of TIICs in Lung Cancer

**FIGURE 3:**
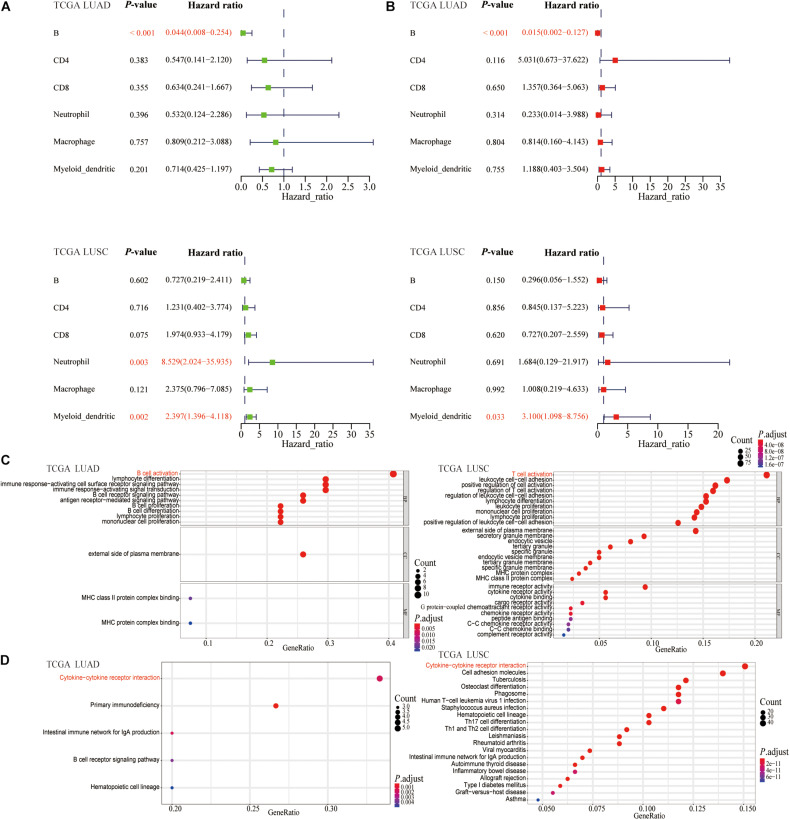
The Cox regression analysis and function enrichment analysis. **(A)** The univariate Cox regression (green) and **(B)** multivariate Cox regression (red) analysis of six types of TIICs in LUAD and LUSC. The vertical dashed line represents HR = 1. B cell was a protective factor for LUAD (HR < 1), whereas DC1 was a harmful factor of LUSC (HR > 1). **(C)** GO and **(D)** KEGG enrichment analysis of 29 genes strongly related to B cell in TCGA-LUAD and 487 genes highly related to DC1 in TCGA-LUSC, respectively. The horizontal axis, gene ratio, represents the proportion of highly related genes contained in the corresponding term to the total highly related genes. The size of the dot indicates the number of highly related genes contained in the corresponding term.

The prognostic landscapes of TIICs in lung cancer were characterized in forest plots. The univariate Cox regression analysis revealed that B cell was associated with good prognosis [hazard ratio (HR) = 0.044, 95% confidence interval (CI) = 0.008–0.254, *P* < 0.001] in LUAD patients, whereas both DC1 and neutrophil were associated with poor prognosis (HR = 2.397, 95% CI = 1.396–4.118, *P* = 0.002; HR = 8.529, 95% CI = 2.024–35.935, *P* = 0.003) for LUSC patients (Figure 3A). The multivariate Cox regression analysis illustrated that B cell was related to good prognosis (HR = 0.015, 95% CI = 0.002–0.127, *P* < 0.001) in LUAD patients, whereas DC1 was related with poor prognosis (HR = 3.100, 95% CI = 1.098–8.756, *P* = 0.033) in LUSC patients (Figure 3B). Therefore, B cell and DC1 were independent prognostic factors for LUAD and LUSC patients, respectively.

### The Functional Enrichment Analysis of Genes Highly Related to B Cell or DC1 in Lung Cancer

To evaluate the biological functions of B cell and DC1 in the occurrence and development of LUAD and LUSC, respectively, in the TCGA-LUAD and TCGA-LUSC transcriptome data, including 19,464 protein-coding genes, we performed the Spearman correlation analysis. Based on the absolute Spearman correlation coefficient greater than 0.6, we obtained 29 protein-coding genes strongly related to B cell in TCGA-LUAD and 487 protein-coding genes highly related to DC1 in TCGA-LUSC. Then, GO and KEGG enrichment analyses were performed by these 29 and 487 genes, respectively. In terms of GO analysis, 29 protein-coding genes were mainly enriched in B cell activation, whereas 487 protein-coding genes were significantly enriched in T cell activation (Figure 3C). In KEGG analysis, Cytokine–cytokine receptor interaction was the most significant pathway of enrichment (Figure 3D). It could be seen that B cell and DC1 abundance, as independent prognostic factors, were closely related to the immune activation status of lung cancer patients. Besides, we inferred, for the prognosis of LUAD patients, that activated humoral immunity played more prominent roles, whereas activated cellular immunity played more significant roles in the prognosis of LUSC patients.

### Expression Profiles of Adaptive Immune Resistance Markers and CD8A and CD8B in LUAD and LUSC

**FIGURE 4:**
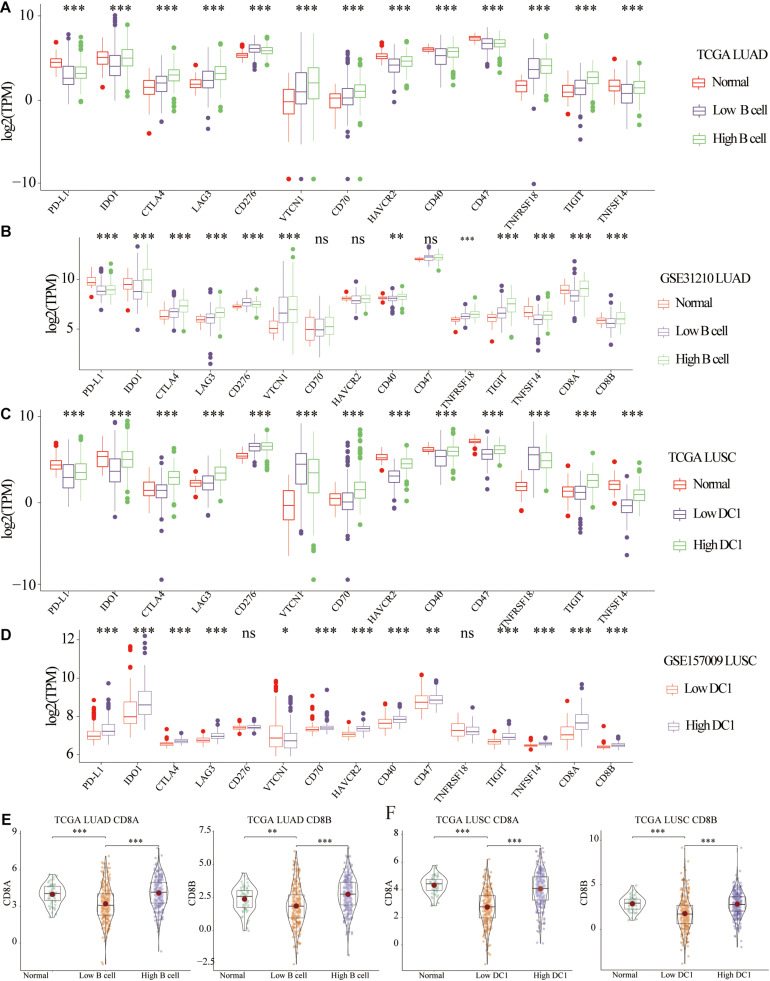
Expression profiles of adaptive immune resistance markers and CD8A and CD8B in LUAD and LUSC. Differential expression of adaptive immune resistance markers among normal, low, and high B cell groups in **(A)** TCGA LUAD and **(B)** GSE31210 LUAD cohorts. Differential expression of adaptive immune resistance markers among normal, low DC1, and high DC1 groups in **(C)** TCGA LUSC and **(D)** GSE157009 LUSC cohorts. Differential expression of CD8A and CD8B in **(B)** GSE31210 LUAD, **(E)** TCGA LUAD, **(D)** GSE157009 LUSC, and **(F)** TCGA LUSC cohorts. ^∗^*p* < 0.05 ^∗∗^*p* < 0.01, ^∗∗∗^*p* < 0.001.

Enrichment analysis revealed that B cell or DC1 abundance, as independent prognostic factor, was significantly related to the immune activation of the TME of lung cancer. Previous research had shown that ICB therapy, mainly targeting the PD-L1/PD-1 axis, had produced a favorable clinical response in cancer patients (Remon and Besse, 2017). Therefore, we hypothesized that LUAD patients with high B cell infiltration or LUSC patients with high DC1 abundance were more suitable for ICB treatment. According to the median abundance of B cell or DC1 in each tumor sample, all tumor samples were classified into high- and low-infiltration groups. In terms of LUAD patients, most biomarkers, including IDO1, CTLA4, LAG3, CD40, TNFRSF18, TIGIT, and TNFSF14, were significantly increased in tumor tissues with high B cell abundance compared with that of low B cell group (Figures 4A,B). For LUSC patients (Figures 4C,D), compared with tumor tissues with low DC1 abundance, PD-L1, IDO1, CTLA4, LAG3, CD70, HAVCR2, CD40, CD47, TIGIT, and TNFSF14 were significantly increased in low DC1 infiltration tumor tissues. However, in LUAD patients, the expression of CD276 was higher in the low B cell group, whereas in LUSC patients, the expression of VTCN1 was higher in the low DC1 group. For LUAD and LUSC patients, the expression level of most adaptive immune resistance markers in high B cell/DC1 infiltration tumor tissues tended to be normal tissues.

In addition, previous studies had proved that CD8A mRNA levels could predict the ICB response (Fumet et al., 2018). CD8A and CD8B expressions were also significantly correlated with cytolytic activity (Rooney et al., 2015). Compared with low B cell group, high B cell group and normal tissues had higher expression of CD8A and CD8B (Figures 4B,D,E). Similarly, high DC1 LUSC and normal tissues had higher CD8A and CD8B expression than low DC1 infiltration tumor samples (Figures 4B,D,F).

### Correlation Between B Cell, DC1, and Immunomodulators (Immunostimulators, MHC, Receptors, and Chemokines)

**FIGURE 5:**
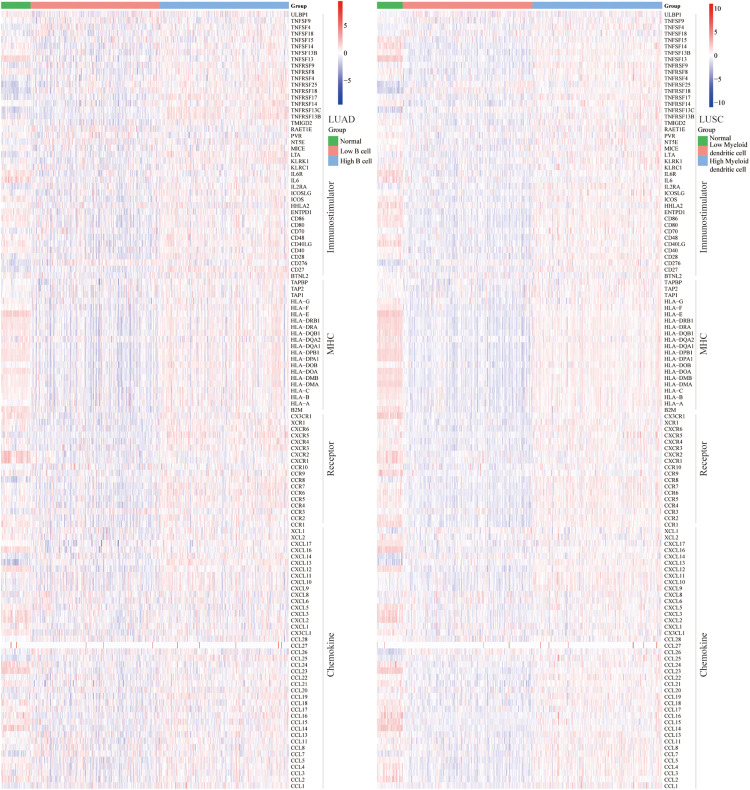
Heatmaps of immunomodulators (immunostimulators, MHC, receptors, and chemokines). Most immunomodulators were upregulated in the high-infiltration group. Each column represents a sample, and each row represents one of the immunomodulators. The expression level of immunomodulators was displayed in different colors, transitioning from blue to red with increasing expression.

To determine the ICB response, we further explored the relationship between abundance of B cell and DC1 and expression of immunostimulators, MHC, receptors, and chemokines. For LUAD and LUSC patients, B cell and DC1 were positively correlated with most immunomodulators (Figure 5). Most MHC molecules in the high B cell/DC1 infiltration group were upregulated, indicating that the high-infiltration group’s antigen presentation and processing capacity were strengthened. These upregulated chemokines and receptors recruited more CD8 T cells, T_H_17 cells, and antigen-presenting cells into the TME of LUAD and LUSC patients. Therefore, we concluded that high B cell/DC1 infiltration shaped an inflamed and immune-activated TME, which was necessary for the success of ICB therapy.

### The Abundance of B Cell and DC1 Was Significantly Related to Immune-Related Pathways

**FIGURE 6:**
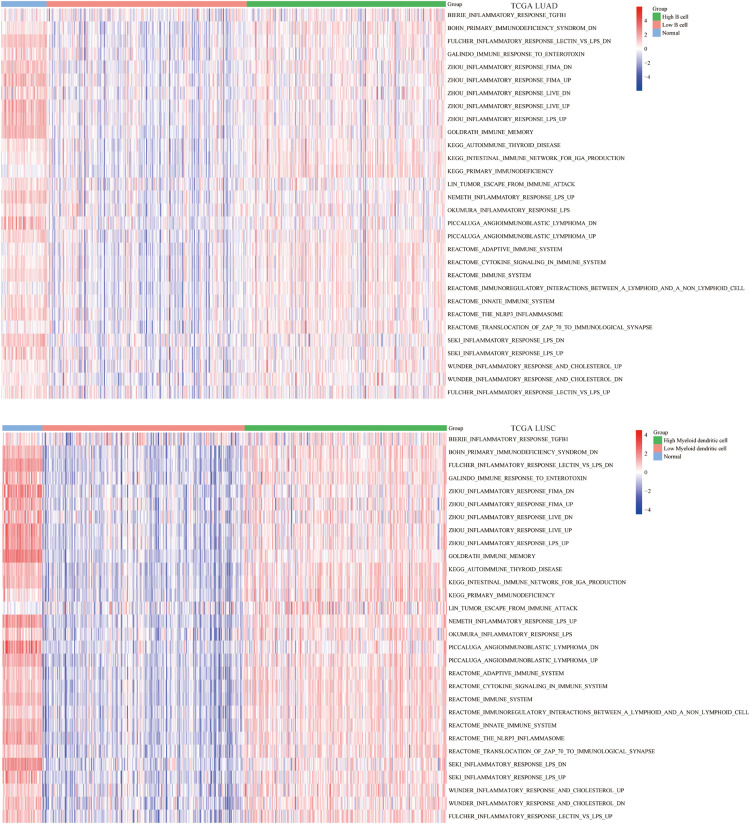
Differences in ssGSEA scores of 30 immune-related pathways among normal, low, and high B cell/DC1 groups in lung cancer. With the increase of B cell/DC1 infiltration, most immune-related pathways were significantly activated.

Apart from single biomarker and immunomodulators, we further studied the correlation between the abundance of B cell and DC1 and immune-related pathways using the ssGSEA algorithm. The enrichment results indicated that in the high B cell/DC1 infiltration group, most immune-related pathways were significantly activated. Besides, the degree of immune activation of high B cell/DC1 infiltration tumor tissues was equivalent to that of normal tissues, and this part of the tumor tissues tended to be closer to normal tissues (Figure 6), which further demonstrated that the high B cell/DC1 infiltration group was immune activated and more suitable for ICB therapy.

### The Abundance of TIICs in Lung Cancer

**FIGURE 7:**
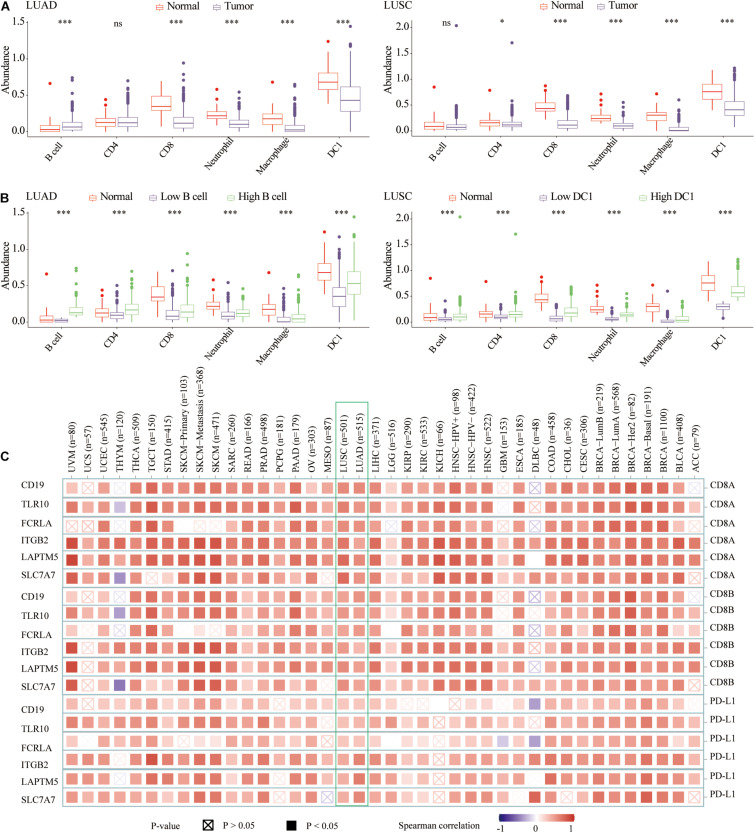
The abundance of six types of TIICs among normal, low, and high B cell/DC1 groups. **(A)** Compared with normal tissues, the abundances of most TIICs were lower in lung cancer tissues. **(B)** For both LUAD and LUSC samples, compared with low B cell/DC1 groups, TIICs all increased significantly in the high B cell/DC1 group. **(C)** In TCGA pan-cancer, hub genes (CD19, TLR10, FCRLA, ITGB2, LAPTM5, and SLC7A7) were significantly related to CD8A, CD8B, and PD-L1. Red represents positive correlation, whereas blue represents negative correlation. The darker the color, the greater the correlation. Solid squares represent *P* < 0.05. *p < 0.05, ***p < 0.001.

Previous studies had shown that the abundance of TIICs could not only predict the survival of cancer patients, but also reflect the efficacy of immunotherapy (Jiang et al., 2018a; Farhood et al., 2019). In terms of infiltration abundance in LUAD and LUSC, DC1 was the most abundant TIICs. Specifically, compared with normal tissues, there was lower CD8 T cell, neutrophil, macrophage, and DC1 abundance, whereas there was higher B cell infiltration in LUAD tissues. For LUSC tissues, the infiltration of CD4 T cell, CD8 T cell, neutrophil, macrophage, and DC1 was lower than that of normal samples (Figure 7A). For both LUAD and LUSC samples, compared with the low B cell/DC1 infiltration group, six types of TIICs significantly increased in the high B cell/DC1 group (Figure 7B). It could be seen that the high B cell/DC1 group was more like “hot tumor” and would benefit more from immunotherapy.

### Correlation Between Abundance of B Cell and DC1 and Clinical Parameters

To judge which lung cancer patients are suitable for immunotherapy from the existing clinical indicators, we analyzed the correlation between abundance of B cell and DC1 and clinical parameters. Combining the TCGA and GSE31210 datasets, we could not draw the same conclusion for which LUAD patients were suitable for immunotherapy. For LUSC of TCGA and GSE157009, female patients had higher DC1 abundance (Supplementary Figure 1), suggesting that female patients would benefit more from ICB therapy. Regarding other clinical parameters of LUAD and LUSC patients, no unanimous conclusions were drawn.

### Correlation With CD8A, CD8B, PD-L1, TME Score, and Highly Related Genes of B Cell and DC1

To facilitate the clinical application of B cell and DC1, in LUAD (TCGA and GSE31210) and LUSC (TCGA and GSE157009) transcriptome data, we performed the Spearman correlation analysis between abundance of B cell and DC1 and 19,464 protein-coding genes, respectively. Among the top 10 genes most related to B cell in TCGA and GSE31210, we screened three shared genes (CD19, TLR10, and FCRLA). Among the top 10 genes most related to DC1 in TCGA and GSE157009, we also captured three shared genes (ITGB2, LAPTM5, and SLC7A7). For convenience, we called these six genes hub genes (Supplementary Table 1). Based on the TIMER2.0, we found that six hub genes were significantly related to CD8A, CD8B, and PD-L1 in the LUAD and LUSC and most other tumor tissues in TCGA (Figure 7C).

### The PPI Network

**FIGURE 8:**
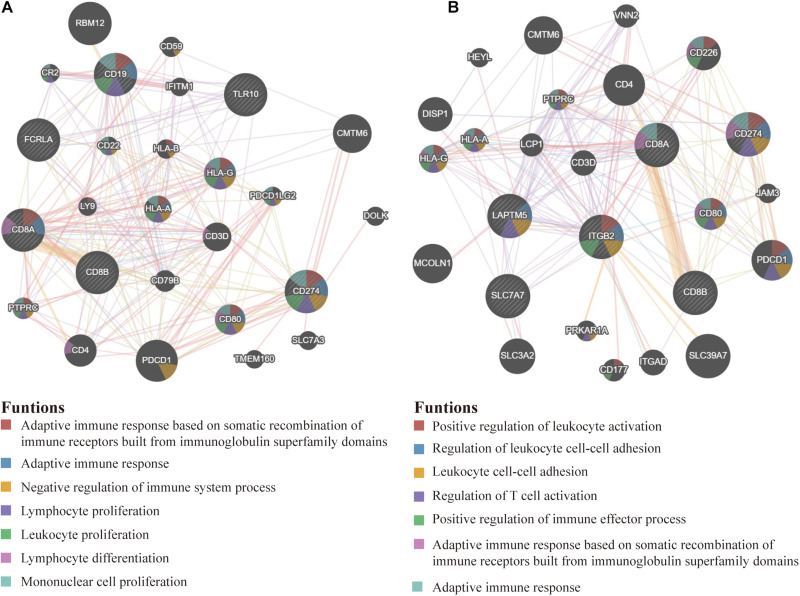
The PPI network. **(A)** The most relevant genes of PD-L1, CD8A, CD8B, CD19, TLR10, and FCRLA are displayed in the PPI network. **(B)** The most relevant genes of PD-L1, CD8A, CD8B, ITGB2, LAPTM5, and SLC7A7 are also shown in the PPI network. Their significant enrichment results are listed below (*P* < 0.05).

On the GeneMANIA website, we further developed two PPI networks, one of which contained 20 proteins most related to PD-L1, CD8A, CD8B, CD19, TLR10, and FCRLA (Figure 8A), and the other contained 20 proteins most related to PD-L1, CD8A, CD8B, ITGB2, LAPTM5, and SLC7A7 (Figure 8B). The functional enrichment results of the above two groups of 23 proteins were related to the activation and proliferation of immune cells. This confirmed that six hub genes were significantly related to the activation of immune system.

### Correlation With the Overall TME Score

**FIGURE 9:**
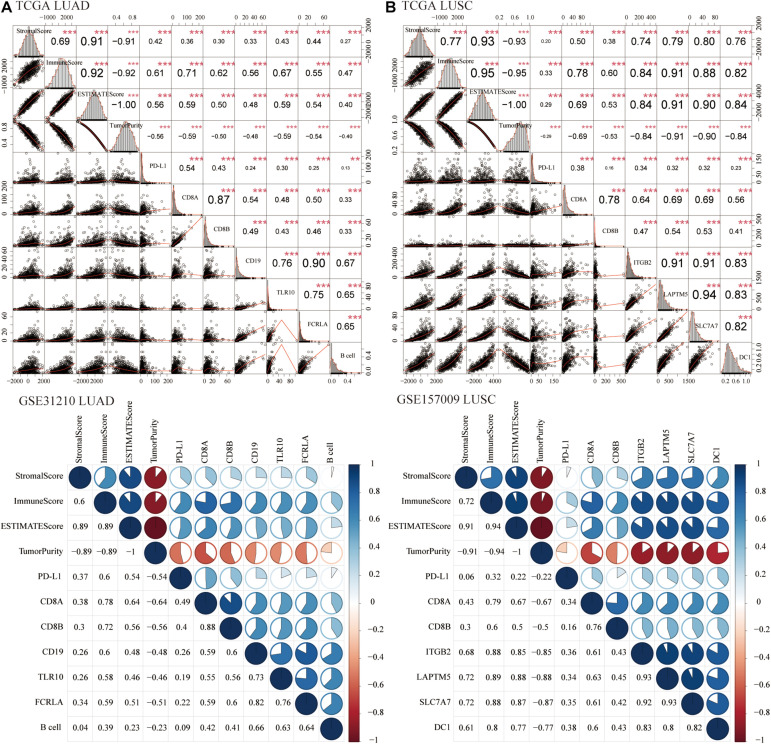
Correlation with the overall tumor microenvironment (TME) score. For LUAD (TCGA and GSE31210) **(A)** and LUSC (TCGA and GSE157009) **(B)** patients, PD-L1, CD8A, CD8B, six hub genes, B cell, and DC1 were significantly positively related to the stromal score, immune score, and ESTIMATE score, whereas they were negatively associated with tumor purity. **P < 0.01, ***P < 0.001.

Growing evidence had indicated that stromal score and immune score could be used as novel biomarkers to predict the prognosis and response to immunotherapy of cancer patients (Jiang et al., 2018b; Lambrechts et al., 2018; Ren et al., 2020). Therefore, we calculated the correlation of six hub genes with stromal score and immune score. For LUAD (Figure 9A) and LUSC (Figure 9B) patients, six hub genes, B cell, and DC1 were significantly positively related to stromal score, immune score, and ESTIMATE score, whereas they were negatively associated with tumor purity, which indicated that lung cancer tissues with high expression of hub genes and abundance of B cell and DC1 were more like “hot tumors,” with lower tumor purity and might benefit more from ICB treatment.

### The Expression Difference of PD-L1, CD8A, CD8B, and Six Hub Genes Between the ICB Non-response (NR) and Response (R) Groups, Five Immune Subtypes of Lung Cancer

**FIGURE 10:**
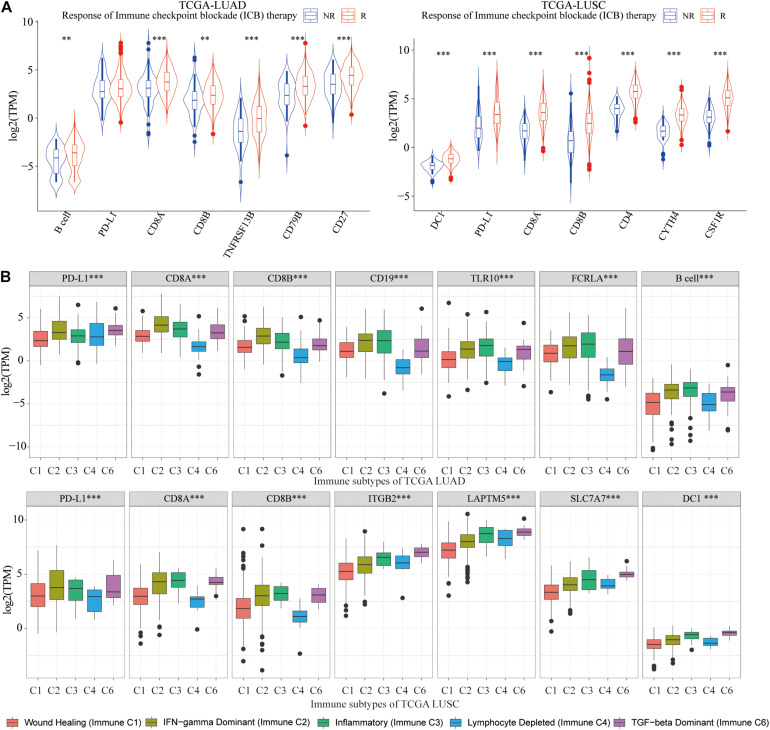
Correlation with ICB treatment and immune subtypes. **(A)** For LUAD, in the ICB response group, B cell abundance, and three hub genes were significantly higher, whereas in LUSC, there were significantly higher abundance of DC1 and expression of three hub genes. **(B)** Among five immune subtypes, immune C2, C3, and C6 had a higher abundance of B cell and DC1 and expression of CD8A, CD8B, and six hub genes. **p < 0.01, ***p < 0.001.

Based on the ImmuCellAI website, lung cancer tissues were classified into the ICB response group (R) and non-response group (NR). We draw the conclusion that for LUAD patients who responded well to ICB therapy, there were significantly higher infiltration of B cell and higher expression of CD8A, CD8B, CD19, TLR10, and FCRLA, whereas in LUSC patients who responded well to ICB therapy, there were significantly higher infiltration of DC1 and higher expression of ITGB2, LAPTM5, and SLC7A7 (Figure 10A). It could be seen that abundance of B cell and DC1 and expression of six hub genes could be used to evaluate the ICB efficacy for lung cancer patients in advance.

As we all know, TCGA-LUAD and TCGA-LUSC patients were classified into five immune subtypes, including wound healing (immune C1), interferon γ (IFN-γ) dominant (immune C2), inflammatory (immune C3), lymphocyte-depleted (immune C4), and transforming growth factor β (TGF-β) dominant (immune C6). Immune C2, C3, and C6 had higher abundance of B cell and DC1, CD8A, CD8B, and hub genes (Figure 10B), which indicated that immune C2, C3, and C6 lung cancer patients were suitable for immunotherapy and would benefit more from ICB therapy.

### The Prognostic Significance of PD-L1, CD8A, CD8B, Six Hub Genes, Age, Gender, and Stage in Lung Cancer

**FIGURE 11:**
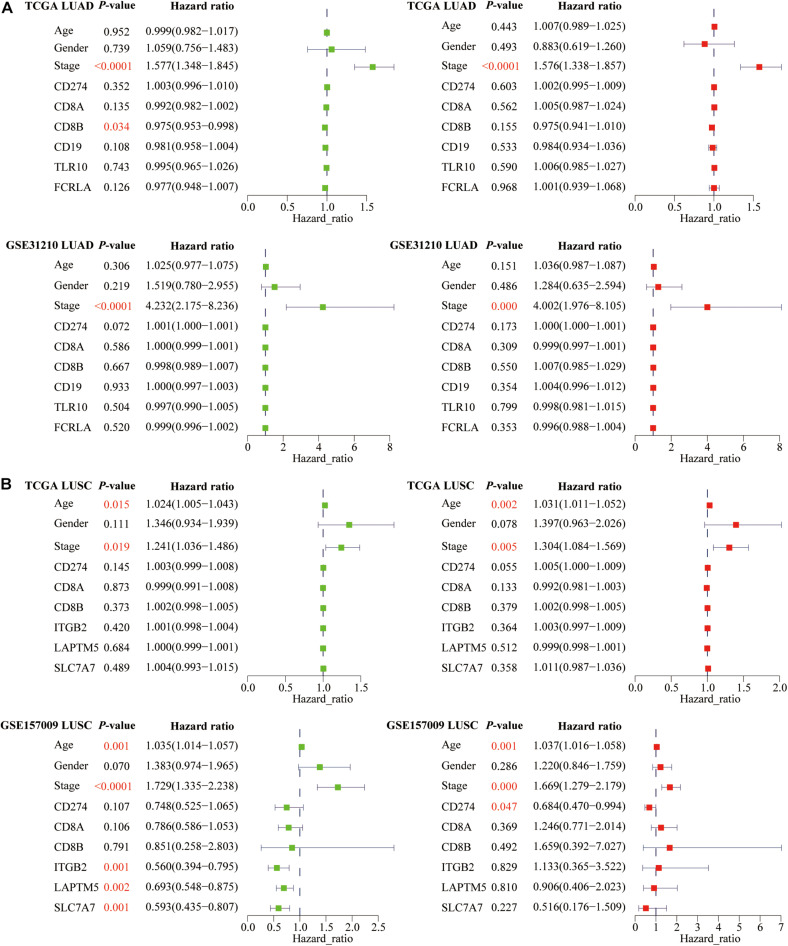
The prognostic significance of PD-L1, CD8A, CD8B, and six hub genes in lung cancer. For **(A)** LUAD and **(B)** LUSC patients, we performed univariate and multivariate Cox regression analyses. Green represents single factor, and red represents multiple factors. *P*-values of independent prognostic factors are marked in red.

In addition to the predictive effect of ICB efficacy, we also studied the prognostic significance of PD-L1, CD8A, CD8B, and six hub genes. Based on the univariate and multivariate Cox regression analyses, we only found that PD-L1 was an independent prognostic factor for LUSC patients (Figure 11B). Besides, among three clinical parameters, stage was an independent factor for LUAD patients (Figure 11A), whereas age and stage were independent factors for LUSC patients (*P* < 0.05) (Figure 11B).

### Identifying Significant and Universally Relevant KEGG Pathways

**FIGURE 12:**
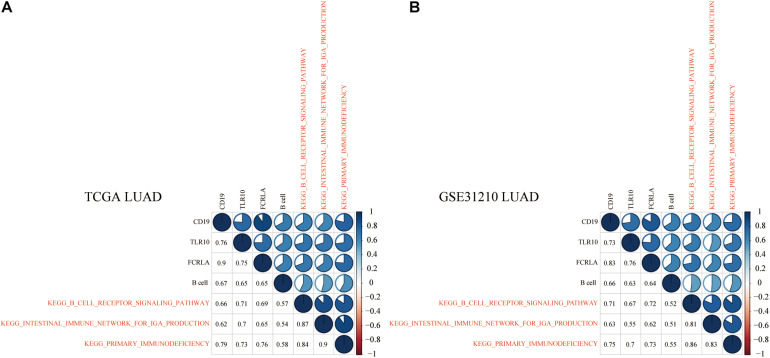
In LUAD tissues of TCGA **(A)** and GSE31210 **(B)**, the above three shared KEGG pathways were highly related to B cell and CD19, TLR10, and FCRLA and are marked in red.

**FIGURE 13:**
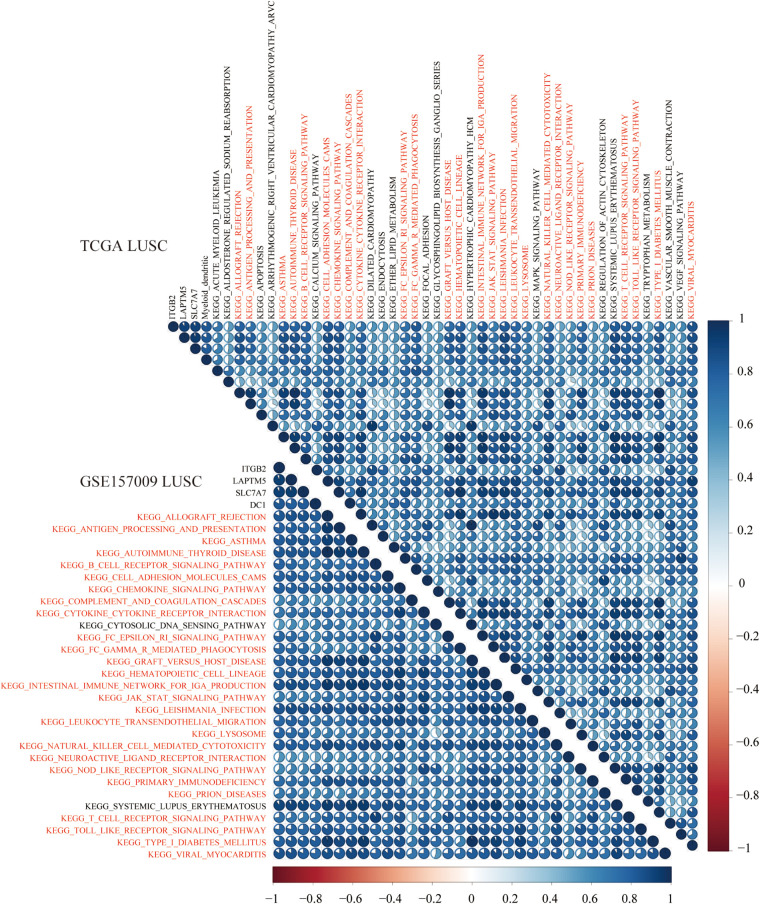
In LUSC tissues of TCGA and GSE157009, the above 27 shared KEGG pathways were highly related to DC1 and ITGB2, LAPTM5, and SLC7A7 and are marked in red.

To explore KEGG pathways that were commonly associated with B cell, DC1, and six hub genes, the ssGSEA algorithm was used to quantify the enrichment score of 186 KEGG pathways in each LUAD and LUSC sample. Taking the Spearman correlation coefficient greater than 0.5 as the threshold, among 186 KEGG pathways, there were 3 and 27 KEGG pathways, which were significantly enriched in LUAD (TCGA and GSE31210) and LUSC (TCGA and GSE157009) cohorts, respectively. Shared enrichment pathways in TCGA and GSE31210 are marked in red (Figure 12). Besides, common enrichment pathways in TCGA and GSE157009 also were highlighted in red (Figure 13).

### KEGG Pathways Related to the Prognosis and the ICB Efficacy Prediction of Lung Cancer Patients

**FIGURE 14:**
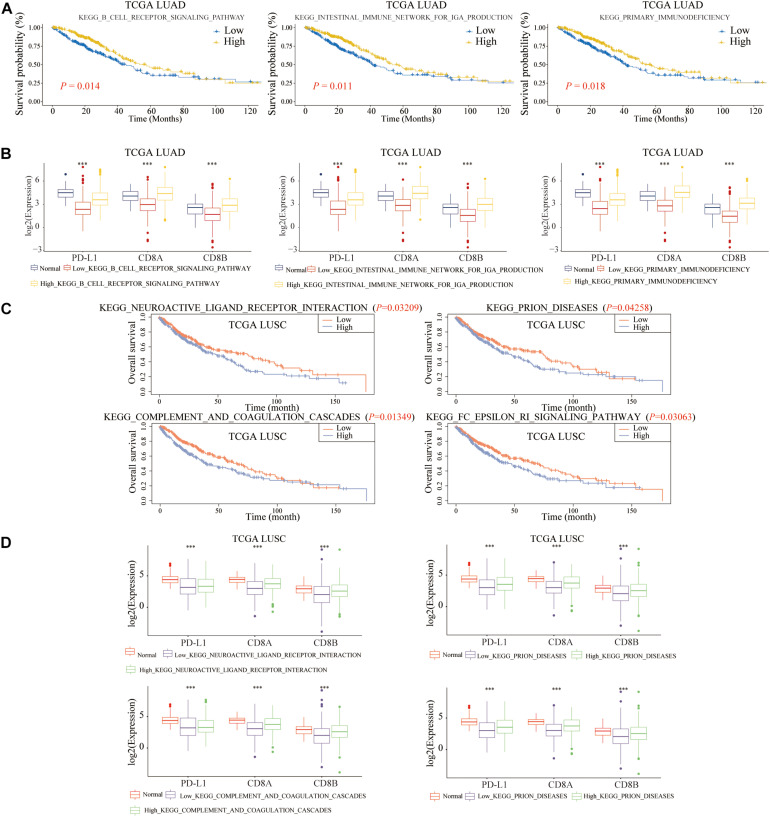
Molecular mechanisms of B cell and DC1 predicting the OS and ICB efficacy of LUAD and LUSC patients. For LUAD patients, the above three KEGG pathways clarified the potential mechanisms of B cell predicting **(A)** the ICB efficacy and **(B)** the OS. The above four pathways elaborated the molecular mechanisms of DC1 predicting **(C)** the ICB efficacy and **(D)** the OS in LUSC patients. ***p < 0.001.

To further explore the molecular mechanisms of B cell and DC1 in predicting the prognosis and the efficacy of immunotherapy simultaneously, based on the median of ssGSEA scores, LUAD and LUSC patients were divided into high- and low-score groups, respectively. As shown in Figures 14A,B, shared KEGG pathways, KEGG B Cell Receptor Signaling Pathway, KEGG Intestinal Immune Network for IGA Production, and KEGG Primary Immunodeficiency, were the molecular mechanisms by which B cell predicted the prognosis and the efficacy of ICB of LUAD patients (*P* < 0.05). For LUSC patients, among the above 27 shared KEGG pathways, KEGG Neuroactive Ligand Receptor Interaction, KEGG Prion Diseases, KEGG Complement And Coagulation Cascades, and KEGG FC Epsilon RI Signaling Pathway were the molecular mechanisms of DC1 that both predicted the prognosis and the ICB efficacy of LUSC patients (*P* < 0.05) (Figures 14C,D). The above seven KEGG pathways were expected to become new immunotherapy targets, and more research was urgently needed.

## Discussion

According to the latest cancer statistics, lung cancer is the second most common cancer, ranking first of cancer-related deaths (Siegel et al., 2020). Therefore, its prognosis and treatment problems need to be solved urgently. With the free opening of public databases, including TCGA and GEO, various prognostic signatures, such as immune-related (Li et al., 2017), glycolysis-related (Zhang et al., 2019a), autophagy-associated (Liu et al., 2019), hypoxia-associated (Mo et al., 2020), metabolism-associated (He et al., 2020), ferroptosis-related (Gao et al., 2021), and TME-associated (Ma et al., 2020) signature, had been established recently. However, previous signatures focused only on the prognosis and did not consider the choice of treatment options for lung cancer patients, especially whether to choose immunotherapy. However, these features focus only on the prognosis, without considering the treatment of lung cancer. In our study, we follow novel findings. B cell was an independent factor for LUAD patients, whereas DC1 was an independent factor for LUSC patients. In many respects, the high B cell/DC1 infiltration shaped an inflamed and immune-activated TME of lung cancer tissues, including adaptive immune resistance markers, CD8A, CD8B, immunomodulators (immunostimulants, MHC, receptors, and chemokines), and immune-related pathways. Besides, tumor tissues in the high-infiltration group were more similar to adjacent normal tissues, whose TME was necessary for the success of ICB therapy. Among five types of immune subtypes of TCGA-LUAD and TCGA-LUSC, IFN-γ–dominant (immune C2), inflammatory (immune C3), and TGF-β–dominant (immune C6) subtypes might be more suitable for ICB therapy. Among 186 KEGG pathways, KEGG B Cell Receptor Signaling Pathway, KEGG Intestinal Immune Network for IGA Production, and KEGG Primary Immunodeficiency were significantly related to infiltration abundance of B cell. Moreover, they were the specific molecular mechanisms by which B cell predicted the ICB efficacy and prognosis of LUAD patients. Similarly, there were four KEGG pathways that clarified the molecular mechanisms of DC1 predicting the prognosis and the ICB efficacy of LUSC patients.

To facilitate clinical application, in the lung cancer transcriptome, including 19,464 protein-coding genes, we extracted the three most relevant genes of B cell (CD19, TLR10, and FCRLA) and DC1 (ITGB2, LAPTM5, and SLC7A7), respectively. Previous immune-related studies on these six genes were as follows.

CD19 is a member of the immunoglobulin gene superfamily and a reliable marker for pre-B cells. The expression of CD19 protein is restricted to B cell lymphocytes. Previous studies have confirmed that it acts as a co-receptor for the B cell antigen receptor complex (BCR). CD19 can decrease the threshold for activation of downstream signaling pathways and for triggering B cell responses to antigens (de Rie et al., 1989; Carter and Fearon, 1992). In short, CD19 molecules play a regulatory role in B cell proliferation and differentiation.

TLR10 is a member of the TLR family that plays a fundamental role in pathogen recognition and activation of innate immunity. Specifically, TLR10 can inhibit the activation and differentiation of monocytes, thereby affecting the DC-mediated adaptive immune response (Hess et al., 2017a). In terms of adaptive immune response, TLR10 is a B cell intrinsic suppressor of adaptive immune responses (Hess et al., 2017b).

There is relatively little research on FCRLA. Fc receptor-like A is selectively expressed in B cells and may be involved in their differentiation and the development of lymphomas (Inozume et al., 2007; Reshetnikova et al., 2012).

Integrin subunit beta 2 (ITGB2) encodes an integrin β chain and is a receptor for ICAM3 and VCAM1. In terms of immune function, ITGB2 regulates the cytotoxicity of natural killer cell (Barber et al., 2004) and is involved in leukocyte adhesion and transmigration of leukocytes including T cells and neutrophils (Ostermann et al., 2002; Bai et al., 2017).

Lysosomal protein transmembrane 5 (LAPTM5) encodes a transmembrane receptor that is associated with lysosomes (Kawai et al., 2014) and plays a crucial role in hematopoiesis (Zhang et al., 2019b). In terms of innate immunity, LAPTM5 protein is a positive regulator of proinflammatory signaling pathways in macrophages (Glowacka et al., 2012). For adaptive immune, LAPTM5 downregulates the level of BCR on the surface of B cells and inhibits B cell activation (Ouchida et al., 2010). LAPTM5 deficiency results in elevated T cell receptor expression on T cells after CD3 stimulation, as well as enhanced T cell responses *in vitro* and *in vivo* (Ouchida et al., 2008).

Solute carrier family 7 member 7 (SLC7A7) encodes the light subunit of a cationic amino acid transporter. Previous studies on SLC7A7 in immunity are as follows. In NSCLC, SLC7A7 is a prognostic biomarker correlated with immune infiltrates (Dai et al., 2021). For T cell acute lymphoblastic leukemia, SLC7A7 inhibits cell apoptosis and promotes cell migration and invasion (Ji et al., 2018). Downregulation of SLC7A7 triggers an inflammatory phenotype in human macrophages and airway epithelial cells (Rotoli et al., 2018).

The functional enrichment analysis of genes significantly related to B cell and DC1 mainly focused on B cell activation and T cell activation in LUAD and LUSC, respectively. Therefore, we concluded that activated humoral immunity might be more critical for the prognosis and the ICB efficacy prediction of LUAD patients, whereas activated cellular immunity might be more crucial for the prognosis and the prediction of ICB efficacy for LUSC patients.

Unlike LUSC patients, for LUAD patients, there was no difference between ICB response and non-response group on the expression of PD-L1. It could be seen that for LUAD patients, PD-L1 could not effectively predict the efficacy of ICB treatment.

## Conclusion

In terms of prognosis, LUAD patients with high B cell infiltration had longer OS, whereas LUSC patients with high DC1 infiltration had shorter OS. In terms of immunotherapy efficacy prediction, lung cancer patients with high B cell/DC1 infiltration, whose TME was inflamed and immune activated, were suitable for ICB therapy. The six hub genes and seven KEGG pathways might become novel potential targets for immunotherapy. Immune C2, C3, and C6 subtypes of lung cancer patients might benefit more from ICB therapy. It is necessary to conduct studies in large cohorts to confirm these findings.

## Data Availability Statement

The datasets presented in this study can be found in online repositories. The names of the repository/repositories and accession number(s) can be found in the article/Supplementary Material.

## Author Contributions

SZ and XL provided direction and concept. XL, XS, and JL analyzed the data and wrote the manuscript. XL and SZ made revisions to the manuscript. All authors have read and approved the final manuscript.

## Conflict of Interest

The authors declare that the research was conducted in the absence of any commercial or financial relationships that could be construed as a potential conflict of interest.

## Publisher’s Note

All claims expressed in this article are solely those of the authors and do not necessarily represent those of their affiliated organizations, or those of the publisher, the editors and the reviewers. Any product that may be evaluated in this article, or claim that may be made by its manufacturer, is not guaranteed or endorsed by the publisher.

## Footnotes

1
https://portal.gdc.cancer.gov/

2
https://www.ncbi.nlm.nih.gov/geo/

3
http://cistrome.dfci.harvard.edu/TIMER/

4
https://www.gsea-msigdb.org/gsea/msigdb/index.jsp

5
http://www.genemania.org

6
http://bioinfo.life.hust.edu.cn/web/ImmuCellAI/

## Abbreviations

TCGA: The Cancer Genome Atlas
GEO: Gene Expression Omnibus
LUAD: lung adenocarcinoma
LUSC: lung squamous cell carcinoma
TIICs: tumor-infiltrating immune cells
DC1: myeloid dendritic cell
OS: overall survival
TME: tumor microenvironment
ssGSEA: single-sample gene set enrichment analysis
ICB: immune checkpoint blockade.
