# Supplementary figures and images for: The Prognosis and Immune Checkpoint Blockade Efficacy Prediction of Tumor-Infiltrating Immune Cells in Lung Cancer
Source: Front Cell Dev Biol. 2021 Aug 3;9:707143. doi: 10.3389/fcell.2021.707143 (PMC8370893; doi:10.3389/fcell.2021.707143)

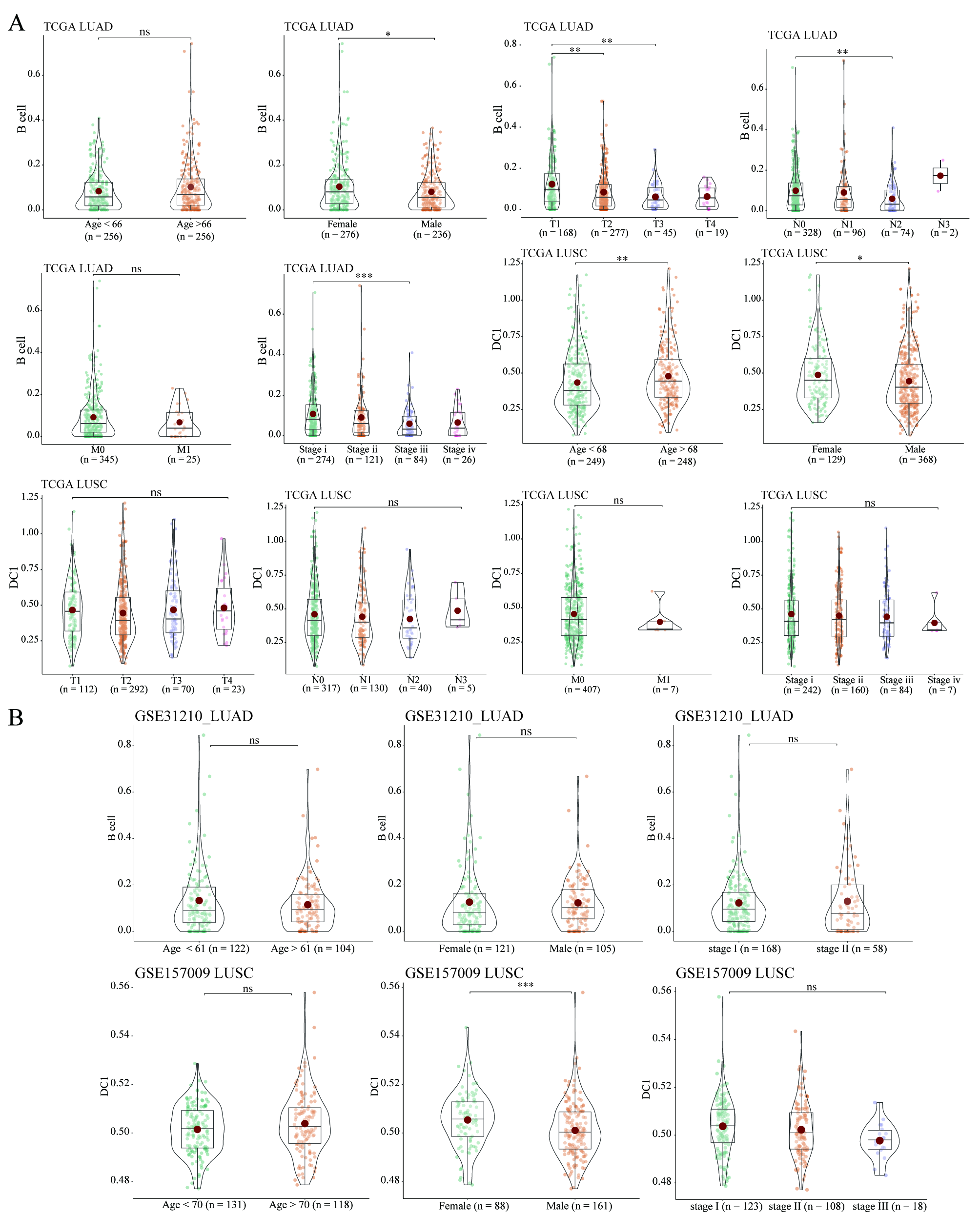

Supplement: Supplementary file 1 [file Image_1.TIF]
