# Supplementary material for: The Prognosis and Immune Checkpoint Blockade Efficacy Prediction of Tumor-Infiltrating Immune Cells in Lung Cancer
Source: Front Cell Dev Biol. 2021 Aug 3;9:707143. doi: 10.3389/fcell.2021.707143 (PMC8370893; doi:10.3389/fcell.2021.707143)
Supplement: Supplementary file 2 [file Table_1.docx]

**Table S1. The top 10 protein coding genes most relevant to B cell and DC1**

| LUAD B cell correlation | | | | LUSC DC1 correlation | | | |
| --- | --- | --- | --- | --- | --- | --- | --- |
| TCGA | | GSE31210 | | TCGA | | GSE157009 | |
| TNFRSF13B | 0.687 | VPREB3 | 0.674 | CD4 | 0.844 | CD86 | 0.828 |
| CD79B | 0.678 | CD19 | 0.662 | CYTH4 | 0.841 | ITGB2 | 0.828 |
| CD27 | 0.672 | FCRLA | 0.642 | CSF1R | 0.840 | CCR5 | 0.817 |
| CD19 | 0.670 | SP140 | 0.637 | ITGB2 | 0.832 | SLC7A7 | 0.816 |
| CLEC17A | 0.665 | CXCL13 | 0.633 | TNFRSF1B | 0.832 | FPR3 | 0.809 |
| BLK | 0.660 | POU2AF1 | 0.630 | LAPTM5 | 0.830 | LAPTM5 | 0.801 |
| P2RY8 | 0.660 | TLR10 | 0.626 | SELPLG | 0.827 | CYBB | 0.792 |
| CD79A | 0.659 | MS4A1 | 0.625 | SPI1 | 0.826 | HAVCR2 | 0.790 |
| TLR10 | 0.648 | CXCR5 | 0.619 | NCKAP1L | 0.822 | FCER1G | 0.788 |
| FCRLA | 0.647 | P2RX5 | 0.612 | SLC7A7 | 0.821 | CLEC4A | 0.781 |
